# A case of occult pancreaticobiliary reflux due to endoscopically confirmed relaxation of the Oddi sphincter

**DOI:** 10.1002/deo2.161

**Published:** 2022-09-26

**Authors:** Fumiya Kataoka, Shin Miura, Kiyoshi Kume, Kazuhiro Kikuta, Shin Hamada, Tetsuya Takikawa, Ryotaro Matsumoto, Mio Ikeda, Takanori Sano, Akira Sasaki, Atsushi Masamune

**Affiliations:** ^1^ Division of Gastroenterology Tohoku University Graduate School of Medicine Miyagi Japan

**Keywords:** biliary tract neoplasms, cholecystitis, congenital biliary dilatation, Oddi dysfunction, pancreaticobiliary maljunction

## Abstract

An otherwise healthy 45‐year‐old woman had been experiencing intermittent right upper abdominal pain for the past 1 year. Computed tomography showed pneumobilia and pancreatic duct emphysema despite a normal duodenal papilla. Magnetic resonance cholangiopancreatography and endoscopic ultrasound confirmed bile duct dilation but without a pancreaticobiliary maljunction. Duodenoscopy detected a slightly sunken, unfixed, and spontaneously enlarged duodenal papilla. During the cholangiogram, the Oddi sphincter was relaxed and the catheter could be easily inserted into the bile duct. Further, no findings suggestive of pancreaticobiliary maljunction were observed, and the contrast medium leaked spontaneously from the duodenal papilla. As biliary amylase level was high, we surmised the occurrence of occult pancreaticobiliary reflux due to relaxation of the Oddi sphincter. However, as there are no guidelines on the management of this condition, we did not offer any treatment. Nevertheless, the patient continued to experience similar symptoms and was retested 1 year later with similar results. As occult pancreaticobiliary reflux was reconfirmed, we suggested that the patient undergo laparoscopic extrahepatic bile duct resection and cholecystectomy, which is the standard treatment for pancreaticobiliary maljunction. Pathological evaluation revealed fibrous thickening of the bile duct wall and chronic cholecystitis, which are typical findings of pancreaticobiliary reflux. Even though pancreaticobiliary reflux is mainly observed in pancreaticobiliary maljunction, it has also been reported in normal patients. Here, we describe a novel mechanism of pancreaticobiliary reflux, namely, a relaxed or defective Oddi sphincter.

## INTRODUCTION

Pancreaticobiliary maljunction (PBM) is defined as the convergence of the pancreatic and bile ducts that are located outside the duodenal wall.^1^, ^2^ It leads to pancreaticobiliary reflux because the papillary sphincter is not affected, and extrahepatic bile duct resection and cholecystectomy are recommended in patients with PBM to prevent the development of the biliary tract cancer.^3^ Furthermore, pancreaticobiliary reflux has been reported not only in patients with PBM but also in individuals with a normal pancreaticobiliary junction including the high confluence of pancreaticobiliary ducts and sphincter of Oddi dysfunction.^4^ Here, we describe a case of occult pancreaticobiliary reflux with endoscopically confirmed relaxation of the Oddi sphincter.

## CASE REPORT

**FIGURE 1:**
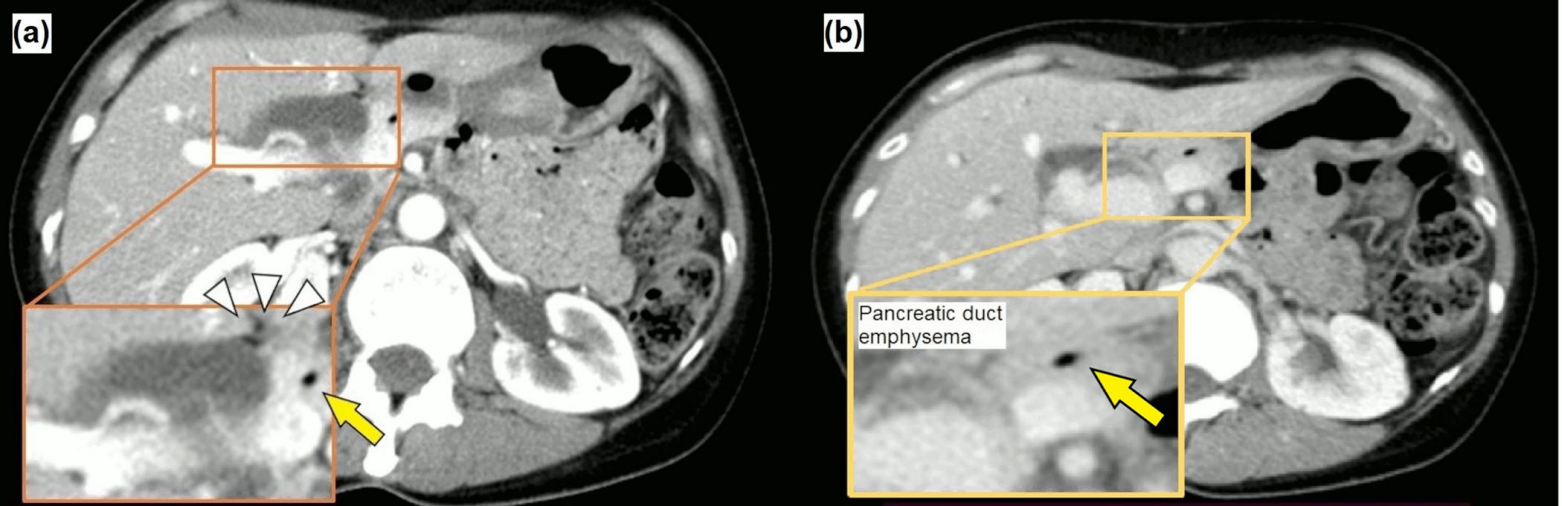
Abdominal computed tomography showed pancreatic duct emphysema (yellow arrow in (a) and (b)) and pneumobilia (white arrowheads in (a)) despite the presence of an untreated duodenal papilla

**FIGURE 2:**
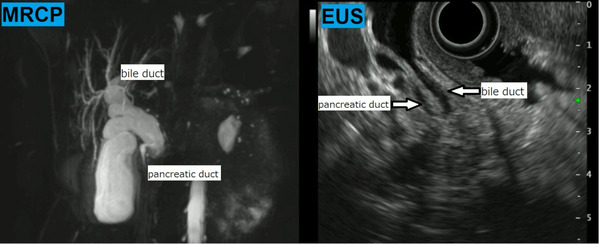
Magnetic resonance cholangiopancreatography and endoscopic ultrasound confirmed bile duct dilation without findings of pancreaticobiliary maljunction

**FIGURE 3:**
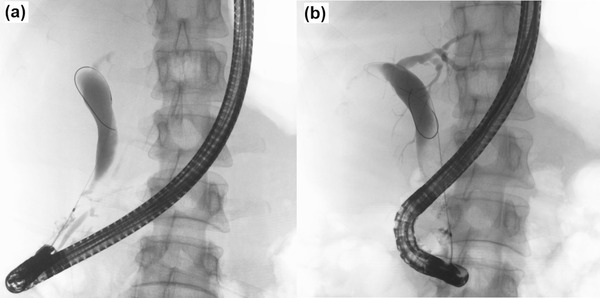
(a) The cholangiogram showed no findings suggestive of pancreatobiliary maljunction and (b) the contrast medium leaked spontaneously from the duodenal papilla

**FIGURE 4:**
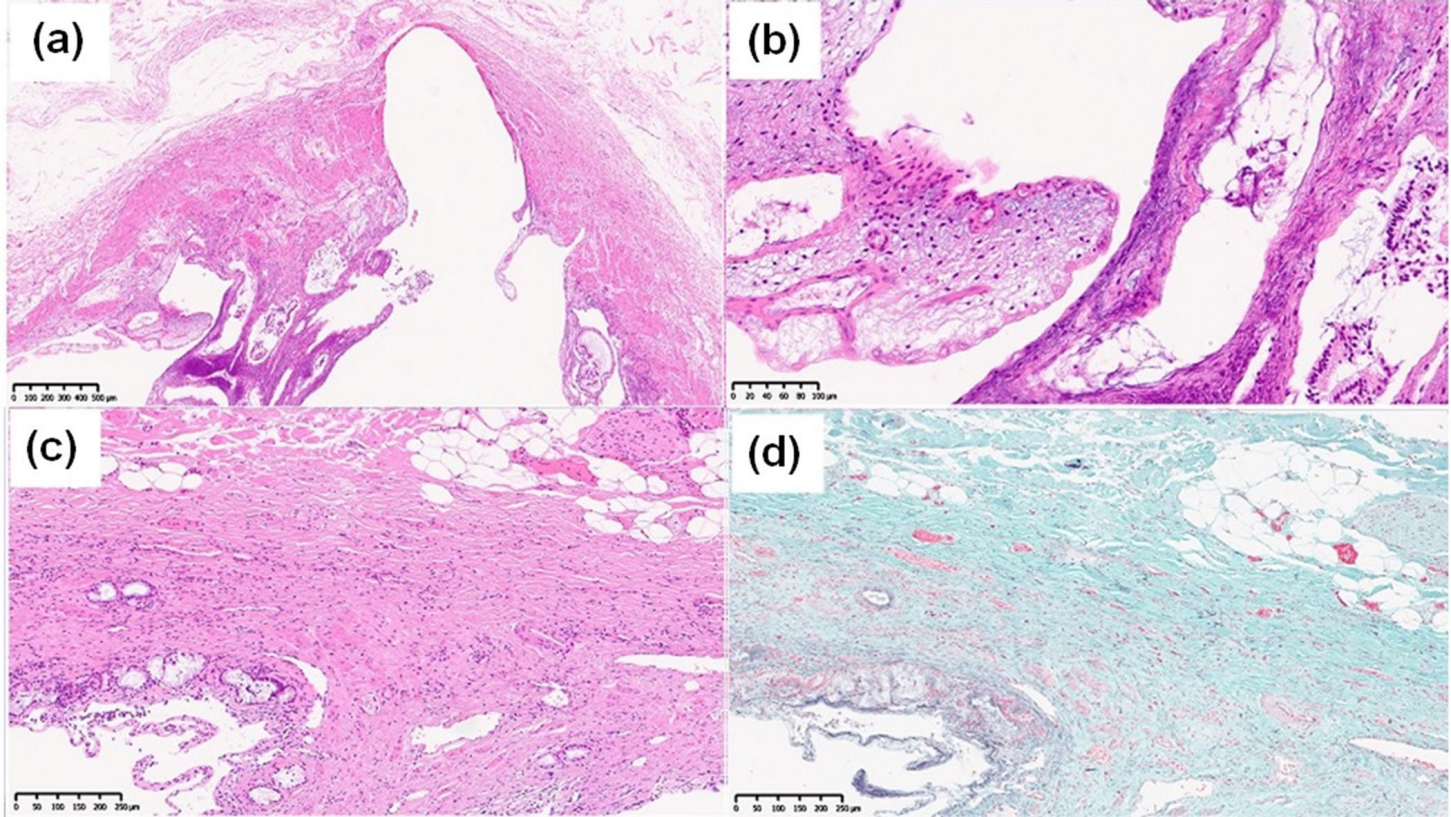
Immunohistological staining with hematoxylin and eosin. (a) The gallbladder showed signs of chronic cholecystitis with thickening of the proper muscular layer (×40), (b) hyperplastic growth of the epithelium (×200), (c) the bile duct showed fibrous thickening (×40), and (d) elastica masson stain revealed more prominent collagen fiber growth

An otherwise healthy 45‐year‐old woman had been experiencing intermittent right upper abdominal pain for the past 1 year. As an abdominal ultrasound at a nearby hospital revealed a gallbladder stone and dilation of bile ducts, she was referred to our hospital for a thorough evaluation of the dilated bile ducts. Her medical and family histories were unremarkable. Blood tests at the first visit showed no elevation in hepatobiliary enzymes, inflammatory response markers, or tumor markers such as carcinoembryonic antigen and carbohydrate antigen 19‐9. Abdominal computed tomography showed pneumobilia and pancreatic duct emphysema, despite the presence of a normal duodenal papilla, along with dilatation of the common bile duct to about 15 mm (Figure 1). Magnetic resonance cholangiopancreatography and endoscopic ultrasonography confirmed bile duct dilation but showed no indications of PBM (Figure 2). However, duodenoscopy revealed a slightly sunken, unfixed, and spontaneously enlarged duodenal papilla, and during the cholangiogram, the Oddi sphincter was relaxed, which enabled easy insertion of the catheter into the bile duct (Video S1). No findings suggestive of PBM and long common duct were seen, and the contrast medium leaked spontaneously from the duodenal papilla (Figure 3). Biliary amylase level was high (3141 IU/L),^5^ and based on imaging findings described above, we tentatively diagnosed the patient with occult pancreaticobiliary reflux due to relaxation of the Oddi sphincter. However, as there are no guidelines for the management of this condition, we did not offer any treatment. The patient continued to experience intermittent right upper abdominal pain and was retested 1 year later. She was prescribed ursodeoxycholic acid (600 mg/day) for 1 year, but it was ineffective in improving abdominal pain. Nevertheless, because the patient had gallbladder stones, we recommended a cholecystectomy and performed endoscopic retrograde cholangiopancreatography) to determine whether extrahepatic bile duct resection was necessary. Both radiological and endoscopic evaluations yielded similar results, i.e., suspicious loss of muscle tissue around the duodenal papilla. As biliary amylase level remained high (Video S2), occult pancreaticobiliary reflux was confirmed, and we suggested that the patient undergo laparoscopic extrahepatic bile duct resection and cholecystectomy, which is the standard of care for PBM. Postoperative pathological analysis of the resected tissue revealed the fibrous thickness of the bile duct wall and chronic cholecystitis, which are typical findings of pancreaticobiliary reflux due to PBM (Figure 4). Her postoperative course was unremarkable and she remains asymptomatic after 1‐year post‐procedure.

## DISCUSSION

Pancreaticobiliary reflux typically occurs in patients with PBM but is also seen in patients with a long common channel or a dysfunctional sphincter of Oddi.^1^, ^4^, ^8^, ^10^ Importantly, many reports state that pancreaticobiliary reflux may be a risk for biliary tract cancer^9^ because it can damage the biliary mucosa and thereby lead to the development of biliary tract cancer. Recent reports have suggested that pancreaticobiliary reflux may occur even when the pancreaticobiliary junction is anatomically normal.^4^, ^6^, ^7^ For example, Itokawa et al. have described the clinical characteristics of patients with high biliary amylase levels and normal pancreaticobiliary junction by categorizing them into high‐level and low‐level groups,^6^ and they state that old age, large bile duct diameter, and bile duct stones are significant risk factors for high biliary amylase levels. Similarly, Sai et al. measured biliary amylase levels in the gallbladder of 64 patients with a normal pancreaticobiliary junction who underwent ERCP for cholecystectomy and report that 6.3% (4/64 cases) of patients had elevated amylase levels and that three of these patients had gall bladder cancer.^4^ These reports indicate that occult pancreaticobiliary reflux can occur under physiological conditions in individuals with a normal pancreaticobiliary junction and that it might be associated with biliary tract cancer.

Furthermore, Itoi et al. have reported that, compared to low biliary amylase levels, high biliary amylase levels led to a significantly higher level of Ki‐67 in the epithelium of the gallbladder and also concluded that occult pancreaticobiliary reflux, especially with high biliary amylase levels, represents an important risk factor for the development of gallbladder carcinoma as well as PBM.^7^

The cause of pancreaticobiliary reflux in the presence of an anatomically normal pancreaticobiliary junction is unknown, but here, we show that relaxation of the Oddi sphincter was responsible for the reflux. The presence of pneumobilia and pancreatic duct emphysema, despite a normal duodenal papilla, was inferred to be due to the duodenal fluid reflux and indicated relaxation of the Oddi sphincter. The duodenal papilla appeared sunken and naturally open as if it were missing musculature, and such a spontaneous opening is not typically observed, even in cases with duodenal diverticulum (Video S3).

One limitation of this case report is that the nature of the tissue near the duodenal papilla could not be verified histologically because a pancreaticoduodenectomy would be necessary, and this was deemed overtreatment.

In summary, we describe a case of occult pancreaticobiliary reflux with endoscopically confirmed relaxation of the Oddi sphincter. This case proposes a novel mechanism for intractable pancreatic juice reflux, and reports of similar cases will help us better understand the mechanism of pancreaticobiliary reflux.

## CONFLICT OF INTEREST

The authors declare no conflict of interest.

## FUNDING INFORMATION

This work was supported by JSPS KAKENHI, Grant Number 21K15915 (to Shin Miura).

## Supporting information




**Supplementary Video S1**: The endoscopic image of the duodenal papilla.

**Supplementary Video S2**: The second endoscopic image of the duodenal papilla. Aberrant motility and spontaneous opening of the duodenal papilla were observed.

**Supplementary Video S3**: A defective sphincter of Oddi was surmised as the cause of the patient's symptoms.
